# Immuno-Hormonal, Genetic and Metabolic Profiling of Newborns as a Basis for the Life-Long OneHealth Medical Record: A Scoping Review

**DOI:** 10.3390/medicina57040382

**Published:** 2021-04-15

**Authors:** Alekandra Fucic, Alberto Mantovani, Gavin W. ten Tusscher

**Affiliations:** 1 Institute for Medical Research and Occupational Health, 10000 Zagreb, Croatia; 2 Department of Food safety, Nutrition and Veterinary Public Health Istituto to Superiore di Sanità, 00161 Roma, Italy; alberto.mantovani@iss.it; 3 Department of Paediatrics and Neonatology, Dijklander Hospital, 1624 NP Hoorn, The Netherlands; g.w.ten.tusscher@planet.nl; 4 Department of General Practice, Amsterdam University Medical Center, 1081 HV Amsterdam, The Netherlands

**Keywords:** OneHealth, newborn screening, environmental health, medical record

## Abstract

Holistic and life-long medical surveillance is the core of personalised medicine and supports an optimal implementation of both preventive and curative healthcare. Personal medical records are only partially unified by hospital or general practitioner informatics systems, but only for citizens with long-term permanent residence. Otherwise, insight into the medical history of patients greatly depends on their medical archive and memory. Additionally, occupational exposure records are not combined with clinical or general practitioner records. Environmental exposure starts preconceptionally and continues during pregnancy by transplacental exposure. Antenatal exposure is partially dependent on parental lifestyle, residence and occupation. Newborn screening (NBS) is currently being performed in developed countries and includes testing for rare genetic, hormone-related, and metabolic conditions. Transplacental exposure to substances such as endocrine disruptors, air pollutants and drugs may have life-long health consequences. However, despite the recognised impact of transplacental exposure on the increased risk of metabolic syndrome, neurobehavioral disorders as well as immunodisturbances including allergy and infertility, not a single test within NBS is geared toward detecting biomarkers of exposure (xenobiotics or their metabolites, nutrients) or effect such as oestradiol, testosterone and cytokines, known for being associated with various health risks and disturbed by transplacental xenobiotic exposures. The outcomes of ongoing exposome projects might be exploited to this purpose. Developing and using a OneHealth Medical Record (OneHealth^MR^) may allow the incorporated chip to harvest information from different sources, with high integration added value for health prevention and care: environmental exposures, occupational health records as well as diagnostics of chronic diseases, allergies and medication usages, from birth and throughout life. Such a concept may present legal and ethical issues pertaining to personal data protection, requiring no significant investments and exploits available technologies and algorithms, putting emphasis on the prevention and integration of environmental exposure and health data.

## 1. Introduction

Major advances in clinical sciences and environmental health provide a large quantity of data for preventive and curative healthcare. Artificial intelligence programs enable the simulation of complex interactions between intra- and intercellular mechanisms which may be used for different purposes, ranging from diagnostics to pharmacology [1,2]; recent developments in predictive toxicology and exposome-related epidemiology also exploit the integrated added value of molecular signalling and computational models [3,4]. Environmental health, occupational health and clinical medicine are often unnecessarily separated, inhibiting communication in overlapping areas. Such knowledge fragmentation may result in a significant loss of data applicability and their incorrect interpretation. Additionally, the environmental and occupational exposure data often form little or no part of medical history taking in clinical settings. The urgent need for bridging and overcoming silos, in particular by including environmental information into clinical medicine, is being highlighted in several contexts, including the research on non-communicable diseases [5] within the planning of local environmental health initiatives [6] and undergraduate public health degree programs [7].

The present paper intends to discuss the importance and feasibility of the inclusion of environmentally associated parameters in newborn screening programmes following the OneHealth principle in order to develop a OneHealth Medical Record (OneHealth^MR^).

## 2. Bridging Silos Management: The OneHealth Concept

When data are siloed, the same information is stored in different databases, leading to inconsistencies between departmental data. For example, if data on the same patient are stored in different systems, these data can become out of sync over time. In contrast with silos’ data and work management, the increasingly popular OneHealth (OH) concept intends to tackle the complexity of health risks at the interfaces between humans and their environments based on collaboration, communication, and coordination across all relevant sectors and disciplines [8]. Although OH-based approaches were primarily intended for zoonotic diseases, they are increasingly exploited to assess environmental health issues [9,10].

Thus, for example, infertility, which has currently reached epidemic levels in developed countries, and places a significant burden on national healthcare resources, illustrates the importance of a comprehensive view into complex multifactorial health conditions. Although the aetiology of infertility appears to be strongly associated with occupational and environmental exposures, there are very limited initiatives in addressing these aspects in the general population education, and in international programs integrating multidisciplinary projects [11,12]. Currently, data are collected in hospitals and/or fertility clinics, gynaecology outpatient clinics and those of general practitioners. In this manner couples do not reap the full benefits of diagnostic data analysis, for efficient medical solutions, due to a lack of data integration. The latter may be crucial as the origin of infertility may be a consequence of environmental and/or transplacental exposure. Only, occupational exposure is included in medical history taking, usually in a non-systematic anecdotical way.

A holistic, OH-based approach in medicine may be integrated and implemented in programs of personalised medicine. Human beings are often not treated as entities living with environmental and occupational exposures, which may include ionising radiation, chemicals and specific food consumption. Recent developments in exposome research have made interesting efforts to integrate social and environmental determinants [13]. Additionally, sex differences and ethnic specificities, for example in metabolism, excretion and sex hormone levels, should receive adequate attention [14].

Sex is a modulating factor of environmental risks, with effects beyond reproductive health: for instance, prenatal exposure has been associated with altered, sex-specific patterns of DNA methylation in male but not in female newborns in South Korea [15]. A recent example for ethnicity impact is its role in adverse neurobehavioral outcomes in children whose mothers were assessed using environmental mixtures methodology; ethnicity was shown to be a contributory factor, together with stress, and other maternal and pregnancy-related factors [16].

Currently, the exposome represents the most ambitious step towards the comprehensive OH appraisal of health determinants, as it intends to include the totality of exposures from a variety of external and internal sources, from conception onward, over a lifetime, and it also encompasses social relations and socio-economic position on health. The exposome might achieve the incorporation of environmental health into personalised medicine, by integrating the individual history of exposures, lifestyles and genetic susceptibilities [17]. On the other hand, the exposome approach may have the problem of gathering a lot of measures on exposure without proper concepts and tools to be associated with health risks and to address interventions as well as the difficulty of modelling the exposome changes during time [18].

## 3. Newborn Screening as the First Step of OneHealth^MR^

Newborn screening (NBS) is a well-established algorithm for the estimation of genetic, hormone-related, and metabolic diseases in newborns. The number of disease entities included in the NBS varies from country to country. In some countries, it is not structurally performed, whilst in other countries, up to 40 and even more medical conditions are screened; in fact technological developments enable a testing panel covering over 50 disorders [19]. Interestingly, NBS is also gaining momentum in countries outside the affluent “western” areas, such as India, particularly for easily diagnosed and treatable conditions such as congenital hypothyroidism [20]. Indeed, as a paramount feature, NBS provides diagnostic information that prompt prevention and/or treatment [21]. A novelty in NBS is the introduction of genome sequencing which may in the future detect several hundred conditions [22]. The prevention of congenital anomalies and developmental deficits is a significant field where evidence-based and feasible prevention can be achieved by integrating environmental health with genetics and lifestyle [23]. During the last decade, several initiatives have been launched to harmonise NBS across Europe [24] but harmonisation is a complex task, requiring variations in concordance with possible specific known national or regional genetic burdens, and will depend on a country’s health protection conditions.

However, not a single test within NBS includes any estimation of the health risks associated with maternal residential or occupational exposure, with the exception of diabetes [25], a disease with critical contribution from the external environment, including not only diet and lifestyle but also xenobiotics [26]. Thus, prenatal exposure to air pollutants, endocrine disruptors or SO_2_, which are associated with pneumonia, immunologic disturbances, metabolic syndrome or infertility in children [26,27,28] are not monitored by healthcare systems, besides mother–child cohorts and biobanks in ongoing exposome projects such as the European Child Cohort Network, established by the Horizon2020-funded LifeCycle Project [29]. Additionally, serum samples, breast milk, cord blood and placenta are not used as a source of significant information on intrauterine exposure and possible health risks. Information from environmental and food sampling can address and target the most appropriate biomarkers of exposure in a given area, according to the OH concept. For instance, the observation of high levels of arsenic (likely of geochemical origin) in an area of Central Italy by the regional agency for environment has prompted dedicated human biomonitoring [30]; indeed, much information can be derived from routine controls undertaken for regulatory purposes, such as those on pesticide residues in foods [31]. Persistent chemicals giving rise to a body burdens can be readily monitored and can serve as a proxy of exposure status, as a cross-sectional measurement. Major examples at the global level involve transplacental exposure to polyfluoroalkyl substances (PFAS) associated with an increased risk for chronic diseases in adulthood, such as allergy and reduced lung function [32,33] and fat-soluble dioxins, polychlorinated biphenyls (PCBs) and flame retardants [34].

The exposome is clearly relevant to community health but also has deep connections with personal habits and history. In fact, drug–exposome interactions are claimed to be a new frontier of personalised medicine [35]. The significance of the association of microbiota in the gastro-intestinal tract with the brain function and immune system is a current focus of research [36,37]. Recently, it was shown that the intake of antidepressant drugs during pregnancy may be linked to an increased risk for childhood gastro-intestinal disturbances in offspring [38]. Additionally, some aspect of nutrition directly interacts with environmental exposures, the first and main example being the protective effect exerted by an adequate iodine status toward endocrine disrupting chemicals targeting the thyroid [39].

Many endocrine disrupting chemicals are considered to have a moderate to low bioaccumulating potential, however, exposure during a vulnerable susceptibility window can cause permanent effects. Example plasticisers such as several phthalates and bisphenol, which alter male reproductive development and mammary development, respectively, upon exposure in utero; these effects, observed in rodent studies, were considered relevant to human health by the European Food Safety Authority [40,41]. Transplacental exposures to endocrine disrupting chemicals at exposure levels found in the general population are associated with possible life-long impact on growth and development [11,12,34]. In addition, thyroid hormones, which have an obvious relevance to foetal growth and neurodevelopment, the “sex steroid hormones” oestradiol and testosterone may be valuable biomarkers of developmental exposures to endocrine disruptors. Recent studies have shown that transplacental exposure to organophosphate pesticides significantly alter the levels of sex hormones in newborns, especially among female newborns [42]. Similar results have been reported for perfluoroalkyl substances [43]. The disturbance of oestradiol levels has been detected in newborns transplacentally exposed to bisphenol A and phthalates [44]. Bisphenol and parabene levels in cord blood have been shown to be inversely associated with testosterone levels in newborns [45].

Both estradiol and testosterone are crucial in prenatal and postnatal brain development. Testosterone has growth-promoting effects on the left and right brain development [46] and disturbances have been associated with an increased risk of autism [47].

Disturbances in cytokine levels in newborns, which may affect immune function and impaired foetal and infant health, were shown to be associated with higher maternal arsenic exposure [48]. Additionally, maternal exposure to volatile organic compounds have influence on the immune status of the newborn child [49]. A recent Chinese study reported that maternal plasma or serum levels of PFAS were associated with altered biomarkers of inflammation in newborns [50]. However, immunological responses to transplacental exposure may be sex-specific. Significant associations between maternal NO2 exposure and increased cord blood concentrations of IL-33 among girls, but not boys, have been reported [51].

## 4. OneHealth^MR^

The life-long collection of data on one’s taxes, residences, occupational history, marriage, bank accounts, and even criminal convictions, are all available in a large number of countries. These data are linked to an identity number and all the data are centrally collated for easy accessibility. On the contrary, medical history has traditionally been segmented, without a common data pool of a person’s allergies, susceptibility, immunosuppression, diagnostic results, ionising radiation or medication history, all of which are crucial for any kind of medical treatment—especially in case of an emergency.

The concept of a health passport has already been implemented in practice. The Health Passport for cardiovascular patients in India [52] and the MyHealth Passport for children, based on a project of the SickKids Good 2 Go Transition Program available in The Hospital for Sick Children, affiliated with the University of Toronto (https://wapps.sickkids.ca/myhealthpassport/, accessed on 14 April 2021) are such examples, though with limited application. The Health Passport idea was also suggested for the follow up of risks associated with life habits and diet (https://www.nice.org.uk/sharedlearning/the-health-passport-helping-everyone-achieve-long-term-health, accessed on 14 April 2021). Health passports may also be used to record COVID-19 vaccination history.

Following such experiences, OneHealth^MR^ is suggested as a concept for the new generation, which should integrate the results of NBS with core information exploiting the experience of exposome projects. The innovativeness of OneHealth^MR^ is in its data panel, which in addition to the current NBS list, also includes residences, the occupation of the mother (and father), biomarkers of exposure tailored on information coming from environmental and food surveillance (thus implementing the OH framework), some relevant nutritional biomarkers (iodine, first) and biomarkers of effect that can be linked with environmental risk factors and indicate potential health disturbances in the future, such as hormone levels and cytokines [53]. The selected biomarkers would not directly indicate diseases, but would rather inform initial perturbations of physiology that can become serious health problems if left unmanaged. However, such changes would be liable to health interventions by reducing exposures at the community, family and personal level, as well as promoting health and awareness behaviours, such as diet and lifestyles.

Additionally, dried blood spots routinely collected and archived in hospitals may be stored for a longer period of time than in current practice and be used for the estimation of intrauterine exposure, whenever such information would be needed during one’s lifetime [54,55]. For example, routinely collected blood samples can inform on the prenatal exposure to specific chemicals, which also is of clinical relevance for later life [56]. More generally, biomarkers’ panels may reflect the mother’s social, personal (lifestyle, diet) and workplace environment during pregnancy; in its turn the mother’s living environment (in its broader meaning) will exert a biological effect on the progeny with possible life-long consequences: examples include the sex-specific impacts on telomere length in newborns [57] or changes in infant microbiota [58]. As in such cases genetic material would be also collected, specific measures should be taken for its preservation and for accommodating the requirements for comprehensive data collection with those for the protection of personal information, including both legal boundaries and public acceptance [59,60].

The data may be part of a health insurance card which will guarantee a common format for analysis and annual upgrading each time a health insurance card is issued. Additionally, this solution can be understood as a transient towards a datum centralised in the cloud. Thus, the data silos’ effect of fragmented pieces of information, which are difficult to assemble, will be prevented. Software which are already available and that can control data are gathered and analysed while data leaks would be avoided due to the diversity in how data are stored. OneHealth^MR^ is a personal database which carries all the data on occupational exposure and clinical data, which is easily approachable and user friendly. In the aforementioned case of infertility, a couple’s data can be simultaneously compared, which improves the efficacy of aetiology, diagnostics and the decision of further medical treatments.

A specific task in such framework is medical data protection, which demands special care and long-term solutions; these must incorporate plasticity to be upgraded and improved without data loss. Platforms which offer such solutions are already available and give support following international medical standards for data protection; this is crucial in the case of patients who are treated in different countries, to eliminate obstacles for telemedicine. Available programs incorporate pseudonymisation and de-identification, which guarantee a stable data architecture within databases. As large datasets may be expected, strategies to prevent bottlenecks must necessarily address both programming and algorithmic issues, always focusing simultaneously on different topics, such as code flexibility, resilience, efficiency and stability.

## 5. Conclusions

Current advances in medicine and biology enable holistic and personalised medicine. Simultaneously, there has been a global increase in the dissatisfaction regarding the general loss of privacy. Telemedicine shows a significant benefit both for patients and physicians. Information flow by networking, application of artificial intelligence and interdisciplinarity accelerate the accumulation and dissemination of knowledge. The most critical question is how to achieve the hybridisation of current technological and biomedical capacities, while protecting one’s privacy at the same time. The suggested OneHealth^MR^ may be a solution for life-long collection of environmental and occupational exposure and clinical data from NBS results throughout life. Such a holistic approach would improve personalised medicine and significantly advance data exploitation. It is crucial that sex hormone levels and selected cytokines are added to NBS data on transplacental exposure following current knowledge on the impact of the environment and intrauterine development on life-long health risks.

## Data Availability

Not applicable.
